# Molecular Detection and Genetic Typing of Hepatitis E Virus in Wild Animals from Slovakia

**DOI:** 10.1007/s12560-025-09657-z

**Published:** 2025-08-11

**Authors:** K. Dudášová, A. Pavlová, B. Kočíková, M. Urda Dolinská, S. Šalamúnová, L. Molnár, L. Kottferová, A. Jacková

**Affiliations:** 1 Department of Epizootiology, Parasitology and Protection of One Health, University of Veterinary Medicine and Pharmacy in Košice, Komenského 73, 041 81 Košice, Slovakia; 2 Clinic of Birds, Exotic and Free Living Animals, University of Veterinary Medicine and Pharmacy in Košice, Komenského 73, 041 81 Košice, Slovakia

**Keywords:** Hepatitis E virus, HEV-3 genotype, Wild boar, Wild ungulates, Zoonosis

## Abstract

Hepatitis E is an emerging zoonosis caused by the hepatitis E virus (HEV) and is recognised worldwide. Wild boars are considered one of the main reservoirs of the zoonotic HEV-3 genotype. However, HEV-3 has also been detected in many other wildlife species. In this study, we investigated 284 liver and muscle tissue samples from wild boars and 107 liver and muscle tissue samples from four different wild ruminant species (red deer, roe deer, European mouflon and fallow deer) across 35 hunting areas in Slovakia. HEV RNA was detected in 14.2% (95% CI 9.8–18.6%) of the liver and 10.5% (95% CI 0.4–20.6%) of the muscle tissue samples from wild boars but in none of the samples from the wild ruminant species. Phylogenetic analysis based on partial ORF1 and ORF2 of the HEV genome revealed that the Slovak wild boar HEV sequences clustered within the zoonotic genotype HEV-3. Depending on their geographical origin, the obtained sequences clustered into three HEV-3 subtypes: HEV-3a, HEV-3i and HEV-3e. Our findings confirm the circulation of HEV in the wild boar population in the Slovak Republic but not in wild ruminant species.

## Introduction

The hepatitis E virus (HEV) is the aetiological agent of typically self-limiting acute viral hepatitis in humans, which is increasingly recognised as an emerging disease (Kamar et al., 2012). Every year, there are an estimated 20 million HEV infections globally, which, according to the World Health Organization (WHO, 2023), leads to a calculated 3.3 million symptomatic cases of hepatitis E. Recent surveillance data from the European Centre for Disease Prevention and Control (ECDC) indicate an unusual increase in HEV infections within the European Union/European Economic Area (EU/EEA) in January 2024 in Belgium, the Czech Republic and Finland compared to the same time period in 2023 (ECDC, 2024). In Finland, 21 interviewed individuals reported consuming mettwurst or salami of various brands during the incubation period. This observation supports the hypothesis that these types of meat products could be a potential source of HEV infection (ECDC, 2024). However, the exact prevalence of HEV infections across EU member states remains undetermined, as reporting of HEV cases to public health authorities is not mandatory in most EU countries.

HEV is a quasi-enveloped, single-stranded, positive-sense RNA virus. Its genome is 6.4–7.2 kb long and consists of three open reading frames: ORF1 (non-structural proteins), ORF2 (viral capsid protein) and ORF3 (multifunctional protein) (Purdy et al., 2022). According to the International Committee on Taxonomy of Viruses (ICTV), HEV belongs to the *Hepeviridae* family, which includes the *Orthohepevirinae* subfamily. The *Paslahepevirus* genus within this subfamily is classified into two species: *Paslahepevirus balayani* and *Paslahepevirus alci* (Purdy et al., 2022). *Paslahepevirus balayani* is further divided into eight HEV genotypes (HEV-1–HEV-8), with subtypes or genetic variants based on the *p*-distances displayed between the whole genome sequences (Smith et al., 2020).

Genotypes HEV-1 and HEV-2 are responsible solely for human infections, causing large waterborne outbreaks in low-income countries (Kamar et al., 2012). Contrastingly, genotypes HEV-3 and HEV-4 are zoonotic, with HEV-3 being the leading cause of HEV infections in developed countries (Kamar et al., 2017). Wild boars (*Sus scrofa*) and domestic pigs (*Sus scrofa domesticus*) are considered the main reservoir species; however, HEV-3 has also been detected worldwide in several deer species, including red deer (*Cervus elaphus*), roe deer (*Capreolus capreolus*) and fallow deer (*Dama dama*) (Reuter et al., 2009; Kubankova et al., 2015; Kukielka et al., 2016; Neumann et al., 2016; Spancerniene et al., 2019; Kozyra et al., 2021; Fonti et al., 2022). The HEV-4 genotype is considered predominant in many Asian countries (China, Japan, Mongolia, South Korea, etc.) but has occasionally been reported in domestic pigs and humans in Europe (Colson et al., 2012; Monne et al., 2013; Smith et al., 2020).Query Genotypes HEV-5 and HEV-6 appear to be limited to wild boars and have only been isolated in Japan (Sridhar et al., 2017). Genotype HEV-7 is zoonotic, with cases detected in an immunocompromised patient and camels in the Middle East (Lee et al., 2016). HEV-8 has been isolated from Bactrian camels in China (Woo et al., 2016).

Foodborne zoonotic transmission of HEV-3 infection via the consumption of pork and game (e.g. wild boar and deer) raw sausages or undercooked meat and meat products has been confirmed by several studies (Li et al., 2005; Masuda et al., 2005; Takahashi et al., 2004). In Europe, cases of foodborne transmission have also been confirmed by identifying identical viral sequences in patients and contaminated leftover food (Renou et al., 2014; Rivero-Juarez et al., 2017). In another study from France, HEV was detected in various food products containing raw meat or liver, such as the delicacy ‘figatelli’ (Pavio et al., 2014). The consumption of undercooked game or pork liver and meat products is common in some European countries, including Slovakia, and is considered an important risk factor for acquiring HEV infection (Pallerla et al., 2021; Pavio et al., 2017).

This study aimed to investigate the presence of HEV in liver and muscle tissue samples from wild boars and wild ruminants hunted for domestic consumption in Slovakia. In addition, HEV-positive samples were phylogenetically analysed to gather information on the genetic diversity of HEV strains present in Slovak game animals.

## Materials and Methods

### Sample Collection

**Fig. 1:**
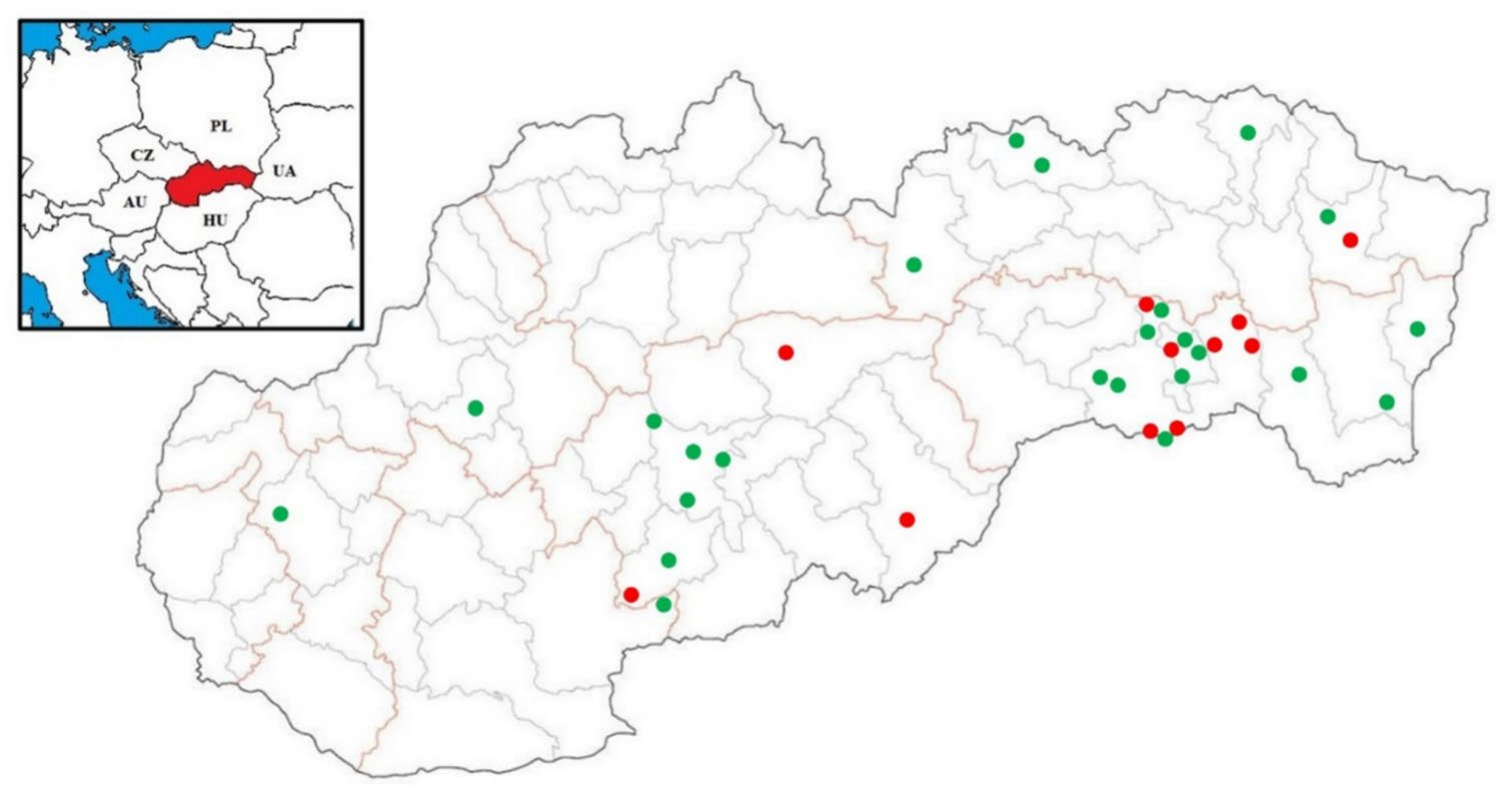
Slovakia map showing the approximate geographical origin of the collected samples (hunting areas). Locations with positive samples are marked with a symbol of red dot, whilst negative samples are marked with a symbol of green dot

In this study, 284 samples (246 liver tissue and 38 muscle tissue samples) from wild boars in the Slovak Republic were analysed (Table 1). Of the 284 samples, 28 were paired liver and muscle tissue samples collected from the same animal at the same time. We also analysed 107 samples (46 liver tissue and 61 muscle tissue samples) from four different wild ruminant species: red deer, roe deer, European mouflon and fallow deer (Table 1). Of the 107 samples, 35 were paired samples collected from the same animal simultaneously. Sampling was carried out between 2015 and 2020 during the regular hunting seasons. Samples were collected from 35 hunting areas across Eastern and Central Slovakia, with a minimum of five samples obtained from each location (Fig. 1). The sample collection was not regulated by the state authorities since HEV is not mandatorily reported in the Slovak Republic. Samples were collected voluntarily under individual agreements with hunters following regional hunting plans approved by the Ministry of Agriculture and Rural Development of the Slovak Republic, which are designed to ensure the sustainable management, maintenance and protection of wildlife populations (Act No. 274/2009 Coll. and Decree No. 344/2009 Coll.). Sample schemes (worksheet) with instructions for the correct sampling were provided to all hunters, and the samples were cut with sterile surgical blades. Approximately 30–50 g of liver tissue and a single block of thigh muscle measuring at least 4 cm × 4 cm × 4 cm were taken and placed in separate collection containers (70 ml).

**Table 1:** HEV detection in liver and muscle tissue samples in different wildlife species hunted in the Slovak Republic

Animal species	Liver tissue		Muscle tissue	
	HEV RNA		HEV RNA	
	Posit/Total (*n*)	Posit (%)	Posit/Total (*n)*	Posit (%)
Wild boar	35/246	14.2	4/38	10.5
Red deer	0/32	0	0/48	0
Roe deer	0/7	0	0/6	0
European mouflon	0/2	0	0/6	0
Fallow deer	0/5	0	0/1	0

The age of wild boars (teeth method; young: < 1 year, sub-adults: 1–2 years, adults: > 2 years) and health status of all wildlife animals was evaluated by inspectors trained by the State Veterinary and Food Administration of the Slovak Republic. Mandatory inspection of wild boars in the Slovak Republic consists of organ pathology during evisceration and sampling for ASF and Trichinellosis in a state veterinary laboratory. The samples were transported fresh at 4 °C or frozen at − 20 °C. Frozen samples originated from locations situated more than 50 km from our laboratory. Upon arrival at the laboratory, the samples were either immediately processed or stored at − 80 °C until the subsequent analysis.

### Nucleic Acid Extraction

Liver samples were homogenised using a mortar and pestle. Briefly, 200 mg of liver tissue was mixed with 1 ml of 0.01 mol/l phosphate-buffered saline (pH 7.4; Sigma-Aldrich, USA). The homogenised samples (20% w/v) were vortexed for three minutes at 351,87×*g* (3000 RPM) using a Multi-Vortex V-32 (Biosan SIA, Latvia) followed by centrifugation at 14,000×*g* for five minutes. A 0.01 mol/l phosphate-buffered saline was used as the negative process control and processed through all analytical stages. A HEV-positive wild boar sample, previously sequenced and verified through BLAST analysis, served as the positive process control. Total RNA was extracted from 200 μl of tissue homogenate (20% w/v) using the TRIzol LS Reagent (Life Technologies, USA). Briefly, 600 μl of TRIzol LS Reagent was added to the samples, followed by vortexing and incubation for five minutes. Subsequently, 120 μl of chloroform (Merck, GmbH, Germany) was added to separate the phases. After incubation, the samples were centrifuged at 12,000×*g* for 15 min at 4 °C, and the aqueous phase was carefully transferred to a new tube. RNA precipitation was carried out by adding 300 μl of isopropanol (Merck, GmbH, Germany), and the RNA pellet was washed with 600 μl of 75% ethanol (Merck, GmbH, Germany). After air drying, RNA was eluted in 20 μl of molecular biology grade water (Merck, GmbH, Germany) and either used immediately or stored at − 80 °C for further analysis. Total RNA from the muscle tissue samples (25 mg) was isolated by the RNeasy Fibrous Tissue Mini Kit (Qiagen, GmbH, Germany) utilising the QIAcube automatic robotic instrument (Qiagen, GmbH, Germany) and processed according to the manufacturer’s protocol. RNA was eluted in 30 μl of RNAse-free water (Qiagen, GmbH, Germany) and used immediately or stored at − 80 °C.

### Molecular Detection

According to Jothikumar et al. (2006), all samples were screened for HEV RNA by real-time RT-PCR with the iTaq Universal Probes One-Step Kit (Bio-Rad Laboratories, Inc., USA). The real-time RT-PCR reaction mixture (20 μl) consisted of 2 µl of isolated RNA, 10 µl of iTaq Universal Probes Reaction Mix, 0.5 µl of iScript Reverse Transcriptase, primers (JVHEV-F, JVHEV-R) at concentration of 250 nM, probe (JVHEV-P) at concentration of 100 nM and molecular biology grade water (Merck, GmbH, Germany). The porcine and bovine house-keeping gene *beta actin* (ACTB) was applied as an internal control to reduce false-negative detections (Lisowski et al., 2008; Wang et al., 2020). For samples from members of the *Cervidae* family, the *glyceraldehyde-3-phosphate dehydrogenase* (GAPDH) gene served as the internal control (Elmi et al., 2020). The RT-PCR reaction was carried out at 50 °C for ten minutes, 95 °C for three minutes and 45 cycles at 95 °C for 15 s, 55 °C for 15 s and 72 °C for 15 s. Real-time RT-PCR was performed on the Bio-Rad CFX96 system (Bio-Rad Laboratories, Inc., USA). Results were analysed with CFX Maestro Software, where samples with threshold cycle (Ct) values lower than 40 were considered positive. The HEV RNA from positive samples was transcripted to cDNA for subsequent analysis. Reverse transcription and nested RT-PCR reactions targeting partial ORF1 and ORF2 regions of the HEV genome were performed according to the protocol described by Jacková et al. (2021). HEV detection was based on the amplification of a 287 bp fragment of the methyltransferase (MTase) domain within ORF1 (Erker et al., 1999) and a 348 bp fragment of the capsid protein gene within ORF2 using primers by Meng et al. (1997). These target sequences were selected from a highly conserved area of HEV genome and enabled comparative analysis with homologous sequences from Slovak pig HEV and other European virus strains.

### Sequencing of DNA and Phylogenetic Analysis

PCR amplicons from selected HEV-positive samples were sequenced in both directions using Sanger sequencing commercially (Microsynth GmbH, Austria). The chromatograms were checked and edited using SeqMan software, and nucleotide sequences were aligned with MegAlign software (Lasergene, DNASTAR, Inc. USA). Based on molecular evolution model tests, phylogenetic trees were constructed employing MEGA X (Kumar et al., 2018). Models with the lowest Bayesian Information Criterion (BIC) values were applied. Maximum Likelihood phylogenetic analysis of the partial ORF1 gene was performed using a General Time Reversible model with gamma distribution plus evolutionarily invariable sites model (GTR + G + I). For the partial ORF2 gene, the Tamura–Nei model with a gamma distribution plus evolutionarily invariable sites model (TN93 + G + I) was used. A total of 1000 bootstrap replicates were used to estimate the support values of individual nodes on the phylogenetic tree. All 41 HEV wild boar sequences from Slovakia obtained in this study were submitted to the NCBI GenBank database under accession numbers PQ650676–PQ650703 for partial ORF1 and PQ657626–PQ657638 for partial ORF2.

### Statistical Analysis

The differences in HEV detection amongst the three age categories of wild boars were analysed using the chi-square test (*χ*2). Values of *P* > 0.05 were considered statistically not significant. HEV prevalence in different age categories was evaluated using 95% confidence intervals (CI). All data analyses were carried out using GraphPad Prism 5 for Windows (GraphPad Software, Inc., USA).

## Results

The presence of HEV RNA was detected in 35 (14.2%, 95% CI 9.8–18.6%) out of 246 liver samples and in 4 (10.5%, 95% CI 0.4–20.6%) out of 38 muscle samples from wild boars (Tables 1 and 2). HEV RNA was present in liver tissue samples in all age categories of wild boars. The most prevalent category was young animals (< 1 year) with 19.2% (95% CI 11.1–27.3%) HEV positivity, then sub-adults (1–2 years) with 13.4% (95% CI 5.9–20.9%) of HEV-positive samples and adults with 8.6% (95% CI 1.9–15.3%) of positive samples (Table 2). The differences in HEV RNA presence in liver tissues across all age categories were not statistically significant (*P* > 0.05, *χ*2 = 3.745), and the differences in muscle tissues were also not statistically significant (*P* > 0.05, *χ*2 = 1.777). HEV RNA was detected in paired liver and muscle tissue samples from five wild boars, with both tissues testing positive in three of these animals. We also analysed liver (*n* = 46) and muscle (*n* = 61) tissue samples from four different wild ruminant species in the Slovak Republic for the presence of HEV RNA. HEV RNA was not detected in any samples from red deer, roe deer, European mouflon or fallow deer (Table 1). The presence of HEV RNA was detected in samples from 11 out of 35 hunting areas analysed within the Slovak Republic (Fig. 1). Amongst these 35 hunting areas, samples were obtained from both wild boar and wild ruminant species in nine. In two of these nine shared hunting areas, HEV RNA was detected solely in wild boars, with no detection in wild ruminants.

**Table 2:** HEV detection in liver and muscle tissue samples of wild boars by the age category of animals

Age category	Liver tissue		95% CI	Muscle tissue		95% CI
	HEV RNA			HEV RNA		
	Posit/Total (*n*)	Posit (%)		Posit/Total (*n*)	Posit (%)	
Young	18/94	19.2	11.1–27.3	2/18	11.1	0–26.7
Sub-adults	11/82	13.4	5.9–20.9	2/11	18.2	0–44.1
Adults	6/70	8.6	1.9–15.3	0/9	0	0–0
Total	35/246	14.2	9.8–18.6	4/38	10.5	0.4–20.6

**Fig. 2:**
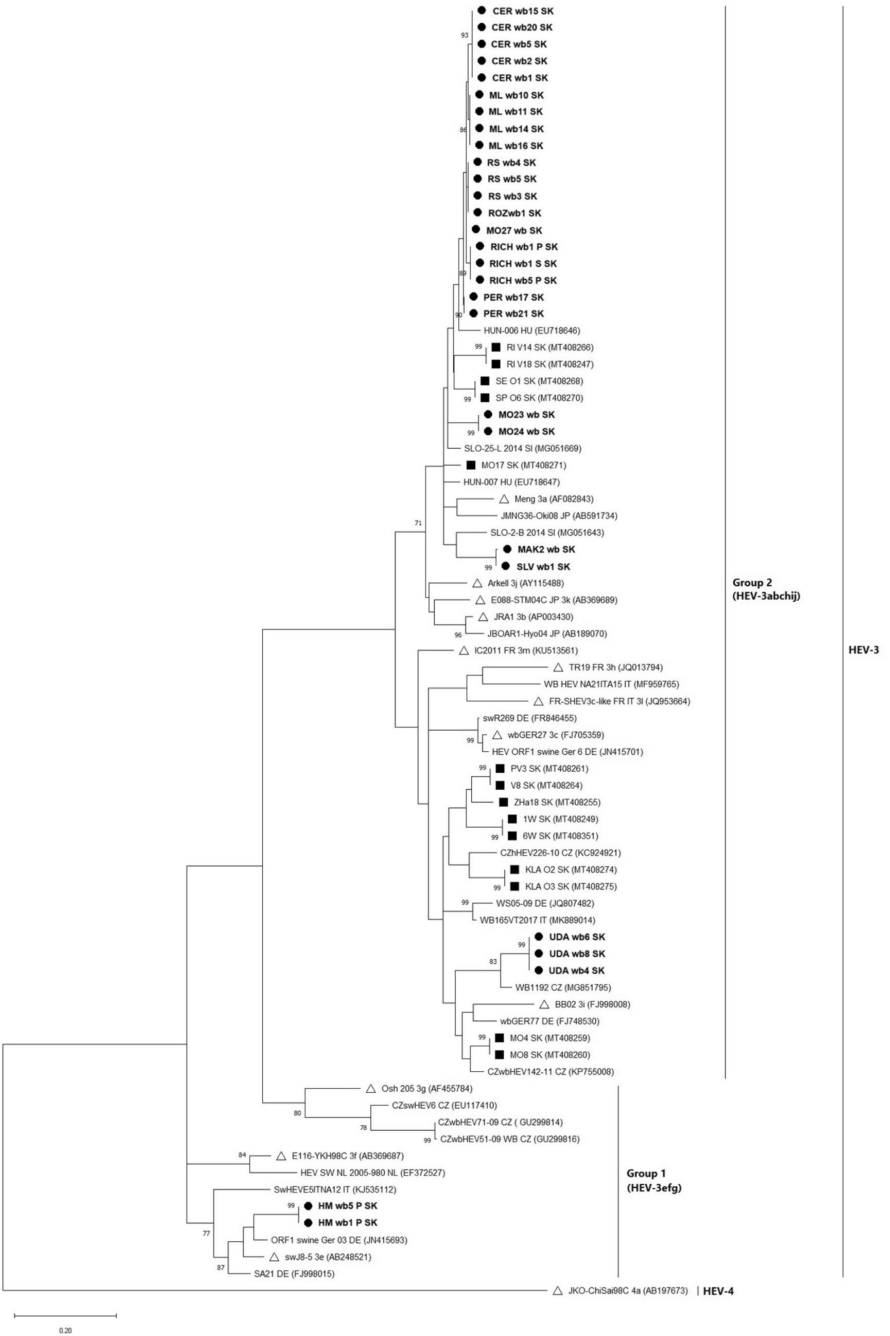
Phylogenetic tree based on the 242 bp nucleotide sequences of the ORF1 fragment of the HEV genome. The tree was constructed using the Maximum Likelihood method and the General Time Reversible model (GTR+G+I) incorporated into MEGA X (Kumar et al., 2018). The sequences from Slovak wild boars are shown in bold and marked with a symbol of black circle, whilst those taken from Slovak domestic swine are marked with a symbol of black square. The reference sequences of the virus strains are marked with a symbol of white triangle and an accession number. All sequences from the GenBank database are marked with an accession number and the country of origin according to ISO 3166-1 alpha-2 codes. The HEV-4 strain was used as an outgroup and the bootstrap values > 70% were shown. The scale bar indicates nucleotide substitutions

**Fig. 3:**
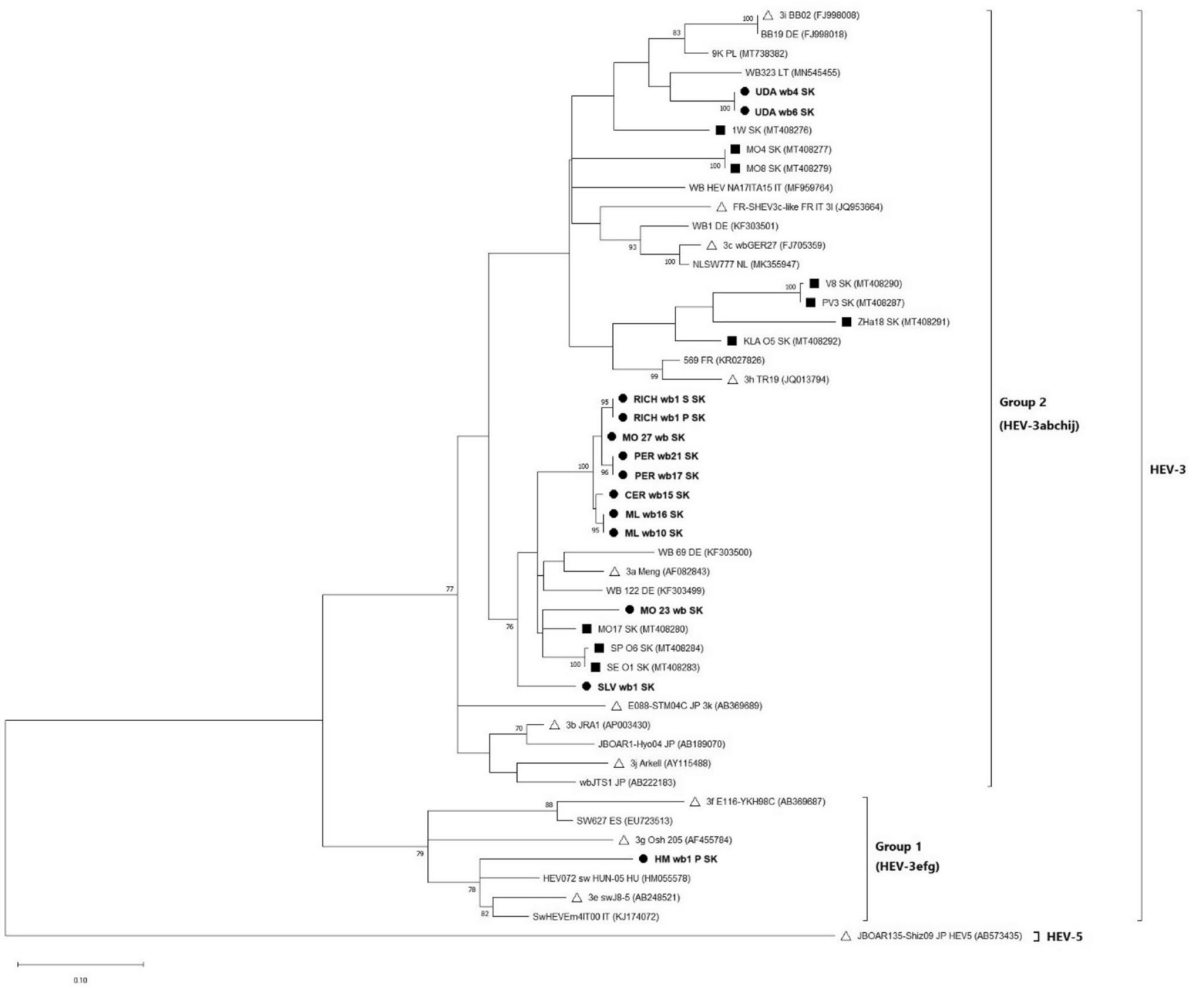
Phylogenetic tree based on the 304 bp nucleotide sequences of the ORF2 fragment of the HEV genome. The tree was constructed by applying the Maximum Likelihood method and the Tamura—Nei model (TN93+G+I) incorporated into MEGA X (Kumar et al., 2018). The HEV sequences from Slovak wild boars are shown in bold and marked with a symbol of black circle, whilst those taken from Slovak domestic swine are marked with a symbol of black square. The reference sequences of the virus strains are marked with a symbol of white triangle and an accession number. All sequences from the GenBank database are marked with an accession number and the country of origin according to ISO 3166-1 alpha-2 codes. The HEV-5 strain was used as an outgroup and the bootstrap values > 70% were shown. The scale bar indicates nucleotide substitutions

For genetic typing of the Slovak HEV wild boar strains, 28 sequences from partial ORF1 (242 bp) HEV gene were selected. Additionally, 13 sequences from the partial ORF2 (304 bp) HEV gene were also selected. Phylogenetic analysis showed the presence of the zoonotic genotype HEV-3 in all obtained Slovak HEV wild boar sequences with mutual nucleotide identity (nt) in the range of 77.6–100% in ORF1 and 78.9–100% in the ORF2 segment of the HEV genome. The sequences detected in this study were grouped according to their geographical origin (hunting area), sharing 100% nucleotide identity and forming separate branches in both the ORF1 (Fig. 2) and ORF2 (Fig. 3) phylogenetic trees. Sequences from liver and muscle tissues obtained from the same animals also exhibited 100% nucleotide identity.

In a more detailed phylogenetic analysis, we found that the obtained sequences clustered into three subtypes within the zoonotic HEV-3 genotype: HEV-3a, HEV-3i (Group 2) and HEV-3e (Group 1), with the HEV-3a subtype being the most represented. Slovak HEV wild boar sequences belonging to the HEV-3a subtype shared 88.0–90.1% nucleotide identity (*p*-distances: 0.099–0.120) in partial ORF1 segment of the HEV genome and 90.8–92.1% (*p*-distances: 0.079–0.092) in partial ORF2 segment with the HEV-3a reference strain Meng (AF082843). Sequences belonging to the HEV-3i subtype shared 88.0% nucleotide identity (*p*-distance: 0.120) in ORF1 of the HEV genome and 90.1% (*p*-distance: 0.099) in ORF2 with the HEV-3i reference strain BB02 (FJ998008). The HEV-3e reference strain swJ8-5 (AB248521) shared 89.6% nucleotide identity (*p*-distance: 0.104) in ORF1 and 88.2% (*p*-distance: 0.118) in the ORF2 segment of the HEV genome with the Slovak HEV-3e wild boar sequences.

## Discussion

Several studies have investigated the presence of the zoonotic HEV-3 genotype in wildlife populations, confirming that this virus actively circulates in European countries (Anheyer-Behmenburg et al., 2017; De Sabato et al., 2020a; Forgách et al., 2010; Kubankova et al., 2015; Kukielka et al., 2016; Pierini et al., 2021). For the first time, this study investigates HEV circulation in wild boar populations and adds new knowledge about the epidemiology of HEV in Slovakia. In the present study, 246 liver tissue samples collected from wild boars were analysed for the presence of HEV RNA, resulting in 14.2% positivity. Different occurrence rates of HEV RNA in wild boars across Europe have been observed in several studies. A similar presence of HEV RNA in liver tissue samples from wild boars was reported in a study from Germany (14.9%) (Schielke et al., 2009). A lower occurrence of HEV in liver samples was reported in studies from Hungary and Croatia (12.2% and 12.3%, respectively) (Prpic et al., 2015; Reuter et al., 2009). In contrast, a higher presence of HEV in wild boar liver samples was observed in Portugal and Italy (25% and 43.6%, respectively) (Beikpour et al., 2023; Mesquita et al., 2016). However, differences in HEV RNA prevalence may be influenced by various factors, such as the type of tissue analysed, the phase of infection at the time of sampling, differences in diagnostic techniques, the sensitivity and specificity of diagnostic protocols, population densities and ecological variations (ecosystem and species diversity) amongst wild boar populations across geographical regions (Caruso et al., 2015; Porea et al., 2018).

Regarding the age categories of wild boars, HEV RNA was detected in liver samples from all categories. The most frequently positive liver samples were from the young category (< 1 year) (19.2%) in comparison to the sub-adults (1–2 years) and the adults categories (13.4% and 8.6%, respectively). Our findings are in correlation with the studies from Croatia (Prpic et al., 2015), the Czech Republic (Kubankova et al., 2015) and Italy (Beikpour et al., 2023), where the samples from the young category of animals were also the most frequently positive for HEV RNA. Moreover, HEV RNA was detected in all age groups. These results suggest that in wild boars, exposure to HEV may also occur later in life (including adulthood). However, in domesticated pigs on farms, HEV RNA is typically detected early in life and shortly after weaning (Leblanc et al., 2007; Di Bartolo et al., 2011; Jackova et al., 2021).

In the present study, 10.5% of the analysed muscle tissue samples from wild boars tested positive for HEV RNA. The presence of HEV in muscle tissue samples was also observed in Germany (Anheyer-Behmenburg et al., 2017; Schielke et al., 2015) and Italy (La Bella et al., 2023). The detection of HEV RNA in muscle tissue samples highlights the potential risk of foodborne transmission of the virus. The consumption of raw or inadequately heat-treated infected meat or meat products (e.g. sausages, stuffing, liver sausages and liver pâté) from wild boars and domestic pigs is the primary route of transmission of HEV in developed countries. Several studies worldwide have confirmed the possible risk of HEV transmission to humans through inadequately heat-treated meat, liver and meat products (Li et al., 2005; Masuda et al., 2005; Rivero-Juarez et al., 2017). The consumption of game meat and sausages is common in Slovakia. Therefore, this study points out a risk of infection for the citizens by confirming the presence of HEV RNA in wild boar liver and muscle tissue.

In this study, phylogenetic analysis confirmed the presence of the zoonotic genotype HEV-3. The HEV-3 genotype is found worldwide and is the most common genotype identified in Europe and America (Treagus et al., 2021). Smith et al. (2020) proposed reference sequences for HEV subtype classification, dividing HEV-3 into Group 1 (HEV-3efg), Group 2 (HEV-3abchij) and the distinct rabbit-derived subtype HEV-3ra. However, new HEV-3 subtypes continue to be identified, making it the most genetically diverse of all HEV genotypes. In Europe, the HEV-3c, HEV-3e and HEV-3f subtypes are the most frequently detected in humans and animals (De Sabato et al., 2020b, 2023; Nicot et al., 2018). Amongst domestic pigs and, particularly, wild boars, the heterogeneity of HEV-3 subtypes is the greatest. For instance, the HEV-3a and HEV-3i subtypes have also been detected in wild boars from Poland (Kozyra et al., 2021), Croatia (Jemersic et al., 2019) and the Czech Republic (Strakova et al., 2018).

The sequences obtained from wild boars in this study clustered into three subtypes (HEV-3a, HEV-3i and HEV-3e) within the HEV-3 genotype. The HEV-3a subtype was the most frequently detected in Slovak wild boar samples. Sequences classified as HEV-3a subtype shared the highest nucleotide identity of 89.7–95.9% (*p*-distances: 0.041–0.103) with two Hungarian wild boar sequences (GenBank Acc. No.: EU718646, EU718647). This similarity could be explained by the migration of wild boars across the Hungary-Slovakia border. The proximity of these animals during feeding, especially in the winter season, as well as faecal contamination of the environment or natural water sources, could also play a role in HEV transmission (Fenaux et al., 2019). However, further investigations are required to better characterize the role of wild boars as a persistent source of HEV infection for animals through direct contact or environmental contamination. The HEV-3a and HEV-3i subtypes were previously confirmed in domestic pig samples from the Slovak Republic (Jackova et al., 2021). The highest nucleotide identity (94.6%) (*p*-distance: 0.054) was observed between two wild boar sequences (PER wb17 and PER wb21) and the sequence MO17 (GenBank Acc. No.: MT408217) from a Slovakian domestic pig inhabiting the same geographical area. Therefore, our results suggest the possible transmission of HEV between these domestic and wildlife swine reservoir animals.

Several studies reported natural or experimental transmission of HEV between domestic pigs and wild boars (Schlosser et al., 2014; Thiry et al., 2017a). The most probable route of HEV transmission between these species is the faecal-oral route, possibly due to close contact in open farming systems or contamination of the environment or water sources. Farmers and farm workers may also contribute to HEV transmission through contaminated clothing and low hygiene standards on farms (Caruso et al., 2017; Krumbholz et al., 2012; Sommerkorn et al., 2017). The active circulation of the zoonotic HEV-3 genotype in Slovak wild boars and domestic pigs without clinical symptoms represents a high risk of infection to public health.

The two sequences classified within the HEV-3e subtype (Group 1), were the most genetically distinct and shared only 77.6–80.5% nucleotide identity (*p*-distances: 0.195–0.224) with HEV-3a and HEV-3i Slovak wild boar sequences. The samples confirmed to contain the HEV-3e subtype were collected from the most distant hunting area in Central Slovakia, whereas the remaining HEV-positive wild boar samples originated from Eastern Slovakia. None of the wild boar HEV sequences from Eastern Slovakia clustered within Group 1 (HEV-3efg), indicating that sequences from different hunting areas are different. The common trade of animals between countries may also contribute to the high diversity of HEV-3 subtypes across different geographical locations. The HEV-3e subtype was also detected in wild boar populations in Hungary, Croatia and Portugal (Forgách et al., 2010; Mesquita et al., 2016; Jemersic et al., 2019).

In this study, none of the 107 muscle and liver tissue samples collected from red deer, roe deer, European mouflon or fallow deer tested positive for HEV RNA. Our findings align with several studies reporting no evidence of HEV infection in wild ruminants. No HEV RNA was detected in wild deer from Italy (Arnaboldi et al., 2021; Serracca et al., 2015) and Croatia (Prpic et al., 2015). Similarly, in Germany, no HEV RNA was detected in liver samples from farmed wild deer species (Trojnar et al., 2020).

On the other hand, HEV infection has been documented in several wild ruminant species across different countries, including Germany (Anheyer-Behmenburg et al., 2017), the Czech Republic (Kubankova et al., 2015), Italy (Di Bartolo et al., 2017) and Hungary (Forgách et al., 2010). However, compared to wild boars, these species consistently exhibit lower HEV RNA prevalences. This suggests that wild ruminants are not true reservoirs of HEV but may acquire the infection incidentally by sharing their natural surroundings with wild boars (Anheyer-Behmenburg et al., 2017; Pavio et al., 2017). Nevertheless, the precise role of wild ruminants in HEV transmission is not yet fully understood (Kubankova et al., 2015; Thiry et al., 2017b). In our analysis, no molecular evidence of HEV was detected in any screened wild ruminants. Our findings raise the hypothesis that these species act as incidental hosts rather than true reservoir species in our country.

However, the surveillance of zoonotic pathogens in wild living animals plays a crucial role in understanding the epidemiology, transmission and dynamics of infectious diseases. Whilst our study provides important insights, there were some limitations. The number of obtained samples, as well as the number of hunting areas, is not sufficient to represent the entire population of wild boars and wild ruminants in our country. However, we have no reason to believe there is any significant deviation from representativeness of our results or the existence of bias. A wider sampling controlled by the state authorities would provide a more comprehensive view of HEV prevalence in wildlife populations in the Slovak Republic. Further analyses are also essential for the evaluation of the potential risks posed by animal products in disease transmission to humans, particularly from a One Health perspective.

## Conclusion

This study confirmed the presence of zoonotic HEV-3 in the liver and muscle tissues of wild boars hunted for domestic consumption in Slovakia. Our findings provide the first documented evidence of the circulation of the zoonotic HEV-3 genotype within the wild boar population in this region of Europe. Phylogenetic analysis revealed that the identified HEV sequences from wild boars clustered into three subtypes: HEV-3a, HEV-3i and HEV-3e. The detection of HEV RNA in wild boar samples highlights a potential health risk associated with the consumption of inadequately heat-treated meat and liver products. Furthermore, our findings suggest a possible risk of HEV transmission from wild boars to domestic pigs (and vice versa) in Slovakia. Finally, this study supports the hypothesis that wild ruminants may serve as incidental HEV hosts, as no molecular evidence of the virus was found in the screened wild ruminant liver and muscle tissue samples.

## Data Availability

No datasets were generated or analysed during the current study.
